# Postweaning Performance of the Agouti (*Dasyprocta leporina*): A Neotropical Rodent with Potential for Domestication

**DOI:** 10.1155/2021/6664656

**Published:** 2021-06-08

**Authors:** Hannah-Marie Samantha Singh, Kegan Romelle Jones

**Affiliations:** ^1^Department of Food Production (DFP), Faculty of Food and Agriculture (FFA), University of the West Indies, St. Augustine Campus, Trinidad and Tobago; ^2^Department of Basic Veterinary Sciences (DBVS), School of Veterinary Medicine (SVM), Faculty of Medical Sciences, University of the West Indies, Mt. Hope, Trinidad and Tobago

## Abstract

This project aimed to observe the effect of different weaning times on the weight gain in agouti (*Dasyprocta leporina*). The goal was to acquire an appropriate weaning time for offsprings. The experiment was performed at the University of the West Indies Field Station Farm (UFS) where animals were divided into four treatment groups, with each treatment group consisted of four animals. Treatment 1 offsprings were weaned at four weeks, treatment 2 offsprings were weaned at three weeks, treatment 3 offsprings were weaned at two weeks, and treatment 4 offsprings were weaned at one week. The offsprings were raised experimentally for their first seven weeks. There was no significant difference (*p* > 0.05) recorded in offsprings live weight and average daily gain (ADG) for treatments 1, 2, and 3. However, offsprings reared in treatment 4 had significantly (*p* < 0.05) less live weight and weight gain in comparison to the other groups at seven weeks. Offsprings in treatment 4 also experienced 50% mortality (2/4 animals died), one animal removed from the experiment due to progressive weight loss, and one offspring remained in the experiment for its duration. The other treatment experienced no loss (0% mortality). Based on the results of the experiment, agouti offsprings should not be weaned at one week due to high mortality and low live weight at the end of seven weeks. Animals can be weaned between 2 and 4 weeks of age with no detrimental effects. Dependent on the level of production, animals can be weaned at 2 or 4 weeks depending on the operators desired litters per year.

## 1. Introduction

In the neotropical region, agouti (*D. leporina*) is one of the most hunted species utilized as a source of meat protein [1, 2]. This rodent has been identified as a selective group of animal that has the potential to be domesticated [3], and some authors have also grouped them as mini-livestock [4]. This rodent is essential as a seed disperser and plays a crucial role in reforestation [5]. This rodent also has a wide range of habitats from savannahs to rainforests [6, 7]. In the wild, these animals prefer to have large burrows which allow them to be concealed from predators [8].

Recently, these animals have been successfully reared in captivity [3]. Agoutis in captivity can be a source of meat which can decrease hunting pressures in the wild, captive animals can also be considered as a form of ex-situ conservation, and finally, captive agouti can be utilized in research. Agouti like most other neotropical animals do not show any form of sexual dimorphism. Identification of sexes must be carried out through physical restraint of the animal. The sex is determined by applying pressure in the pubic region. In male, the penis will protrude, but in female, any protrusion will be absent [3].

There has been some debate on the dietary behaviour of agouti. Some authors have stated that this animal is a frugivore which consumes fruits, nuts, and seeds [9–13]. However, recently, the zoophagic behaviour of this animal has been highlighted [14–17]. Finally, due to the variability in this animal's diet due to environmental conditions, some researchers have classified them as opportunistic omnivores [18]. Agouti is a rodent that possesses a large caecum and practices both coprophagy and caecotrophy [19]. When consuming feed, these animals adopt a sitting position and manipulate its feed with its forelimbs [9]. The preferred feed particle size was found to be 17.5 × 25.4 mm if a pellet for agouti was to be manufactured [20].

Preliminary research was performed on the raising of agouti offsprings postpartum, and one week was found to be the ideal weaning age of the agouti without any detrimental effects [21]. However, Mohammed et al. [21] failed to analyse the dietary constituents of the feed given to agouti in captivity. In the research, mentioned animals were fed on locally available crops, fruits, and forages, but the nutritional content of these feedstuffs were unknown. Thus, this experiment was performed to obtain the optimal weaning age for agouti with animals fed a standard diet with known nutritional constituents.

## 2. Methodology

### 2.1. Research/Experimental Design

This experiment was carried out using four treatments. Each treatment consisted of four animals. In treatment 1 (T1), offsprings were weaned at four weeks, T2 offsprings were weaned at three weeks, T3 offsprings were weaned at two weeks, and T4 offsprings were weaned at one week. Offsprings were weighed weekly postweaning until seven weeks of age. Agouti weights were taken using a digital scale. At the end of the seven weeks, the offspring' final weight was compared and analysed. On a daily basis, the animals were monitored for any clinical signs of ill health which would include lethargy, dehydration, and weakened demeanour.

### 2.2. Animal Housing and Management

The agoutis were housed in an intensive production system. Pregnant females are housed in individual cages until parturition. Offsprings were removed at different ages based on the treatment group assigned, and mothers were subsequently returned to the breeding units. The agouti unit is located at the University Field Station (UFS). It has an open-sided housing system and is arranged in a southern direction. Offsprings were held in cages measuring 0.61 m × 0.61 m *x* 0.41 m inches postweaning. Offsprings were fed daily with rabbit ration (Mastermix ®) and given water ad libitum. The proximate analysis of the rabbit ration is given in Table 1. All treatments were fed with this standard ration. Each animal was given 50 g DM of feed.

### 2.3. Data Analysis

Data were analysed using a completely randomized design with different diet treatments against time using SPSS (20) one-way ANOVA. Repeated measures analysis was performed to test the difference between treatment groups. A significance level of *p* < 0.05 was used throughout the experiment.

## 3. Results

**Figure 1:**
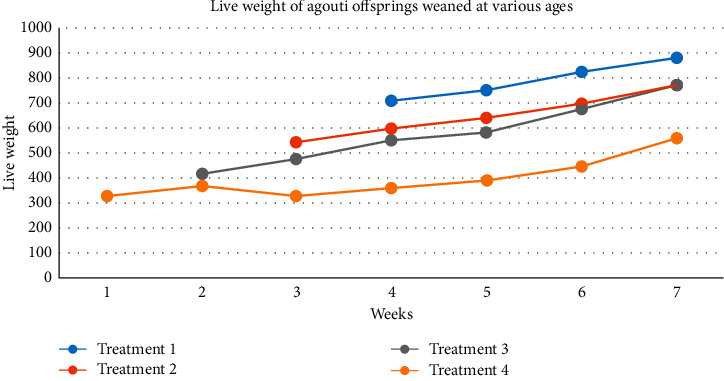
Live weight changes of agouti (*D. leporina*) offsprings at different ages of weaning.

The analysis of data revealed that there was no significant difference (*p* > 0.05) in the live weight between treatments one to three throughout the experiment. Treatment 4 where animals were weaned at one week had significantly lower (*p* < 0.05) live weight compared to other three groups (Table 2, Figure 1). At the end of the seven weeks, animals weaned at one week had the lowest (*p* < 0.05) final bodyweight (560 g), weight gain (320 g), and average daily gain (6.53 g/day) in comparison to animals weaned at two, three, and four weeks. Animals were offered 50 g per day, but the daily intake of the animals were not recorded. However, all animals were offered the same amount of feed.

There was no significant difference between offsprings that were weaned between two and four weeks. Numerically, animals weaned at four weeks had the highest final weight at seven weeks (877 g), weight gain (657 g), as well as average daily gain (13.4 g/day) (Table 3). The average daily gain for each treatment was 13.4 g/day (T1), 11.35 g/day (T2), 9.73 g/day (T3), and 6.53 g/day (T4). Offsprings weaned at four weeks had the highest average daily gain numerically, but statistically there was no significant difference between offsprings weaned at four, three, and two weeks (Table 3).

Offsprings that were weaned at four, three, and two weeks experienced no mortality during the study. However, 50% mortality was recorded when animals were weaned at one week. Also, one animal in treatment four was removed due to signs of illness such as rapid weight loss and lethargy. In summary, animals that were weaned at one week had the least favourable results numerically and statistically (*p* < 0.05). Also, weaning at one week was detrimental to some animals in that group.

## 4. Discussion

The aim of this study was to determine the optimum weaning age for the agouti (*D. leporina*). The analysis of the data revealed that animals weaned at four weeks had the best results numerically but were not statistically different from offsprings that were weaned at two and four weeks. Mohammed et al. [21] conducted a similar experiment, but he recorded higher average daily gains than what was found in this experiment. In that experiment, weaning ages of one, two, three, and six weeks were chosen. The average daily gains were 18.7 g/day (offsprings weaned at one week), 18.5 g/day (weaned at two weeks), 18.4 g/day (weaned at three weeks), and 16.1 g/day (weaned at six weeks) [21]. Differences that were seen can be attributed to the length of the study. Mohammed et al. [21] conducted the experiment for twelve weeks, whilst this experiment was much shorter (seven weeks). In this experiment, the diet used to feed the animals was standardized and its nutritional content was known based on proximate analysis. However, Mohammed et al. [21] used a variety of local feed ingredients and also supplemented with concentrate, but the author failed to analyse the feedstuff that was used in the experiment which could have brought about variability.

In this experiment, mortality was only experienced in animals that were weaned at one week of age. This was in disagreement with results stated by Mohammed et al. [21] that recorded zero mortality in animals weaned at one, two, three, and six weeks. Authors concluded that agouti can be weaned at one week without any detrimental effect [21]. However, the results obtained from this study showed that it is detrimental to wean animals at one week, and it is more feasible to wean animals at two, three, or four weeks of age. Agouti produces precocious young that have opened eyes, teeth, hair, and nails. The mother does not play a major role in nutritional role in agouti, but the mother does demonstrate foraging behaviour that the young learns [22]. However, if animals are weaned too early, these behaviours may not be noticed by the adult, resulting in animals which experience weight loss and reduced growth and mortality as seen in this study.

Agouti can be safely weaned between two and four weeks of age. The age that can be chosen is dependent upon the desired litters per year. The gestation period of agouti has been recorded as 103 days [23] and 104 days [24]. If three litters per year are required from the female, then offsprings can be weaned at two weeks. However, if two litters a year are desired, offsprings can be weaned at four weeks. This research does highlight the fact that the nutritional requirement for growing agouti should be known. Future work should be conducted on the requirement of protein, energy, and fibre in the feed for the juvenile animal. In closing, agouti produces precocious young; however, the digestive tract may not be functionally efficient to utilize solid feed at one week of age. Therefore, based on the results presented, the minimum weaning age of the agouti should be at two weeks.

## 5. Conclusion

The experiment showed that agouti offsprings can be weaned as early as two weeks without any detrimental effects. There was no significant difference in live weight, weight gain, and average daily gain (ADG) (*p* > 0.05) between offsprings weaned at two, three, and four weeks. Animals that were weaned at one week had significantly lower (*p* < 0.05) final weight at seven weeks, weight gain, and average daily gains in comparison to offsprings in other treatment groups. Weaning animals at one week resulted in high mortality (50%) and reduced performance.

## Figures and Tables

**Table 1:** Proximate analysis of the rabbit ration (Mastermix ®) used in all treatment groups.

Feed constituents	
Dry matter (%)	88.39
Crude protein (%)	17.03
Ether extract (%)	5.49
Crude fibre (%)	5.54
Ash (%)	6.92

**Table 2:** Live weight (*g*) of agouti (*D. leporina*) offsprings at various weaning ages.

Weeks	T1	T2	T3	T4	*P* value
1	—	—	—	325	—
2	—	—	415	365	0.12
3	—	543^a^	475^a^	325^b^	0.03
4	705^a^	596^a^	551^a^	360^b^	0.03
5	752^a^	640^a^	581^a^	390^b^	0.02
6	822^a^	693^a^	673^a^	445^b^	0.04
7	877^a^	771^a^	772^a^	560^b^	0.03

a, b indicate the means in the same column that had different superscripts are significantly different (*p* < 0.05). T1, offsprings weaned at four weeks; T2, offsprings weaned at three weeks; T3, offsprings weaned at two weeks; T4, offsprings weaned at one week.

**Table 3:** Final live weight and average daily gain for the offsprings weaned at different times.

Parameters	T1	T2	T3	T4	*P* value
Average birthweight (g)	220	215	295	240	—
Final weight (*g*) (@ 7 weeks)	877^a^	771^a^	772^a^	560^b^	0.03
Weight gain (g)	657^a^	556^a^	477^a^	320^b^	0.04
Average daily gain (ADG) (g/day)	13.4^a^	11.35^a^	9.73^a^	6.53^b^	0.02
Average litter size	1.5	1.25	1.5	1.5	0.65

a, *b* indicate means in the same column that had different superscripts are significantly different (*p* < 0.05). T1, offsprings weaned at four weeks; T2, offsprings weaned at three weeks; T3, offsprings weaned at two weeks; T4, offsprings weaned at one week.

## Data Availability

The data used to support the findings of this study are included within the article.
